# Supplementary material for: A high-resolution mRNA expression time course of embryonic development in zebrafish
Source: eLife. 2017 Nov 16;6:e30860. doi: 10.7554/eLife.30860 (PMC5690287; doi:10.7554/eLife.30860)
Supplement: Supplementary file 6. [file elife-30860-supp6.zip › biolayout-clusters-files/Cluster201-genes.html]

Cluster201


# Cluster201: Genes

| Ensembl ID | Gene Name | Chr | Start | End | Biotype |
| --- | --- | --- | --- | --- | --- |
| ENSDARG00000032405 | ENSDARG00000032405 | 4 | 76590519 | 76594730 | protein\_coding |
| ENSDARG00000102105 | dad1 | 2 | 52047829 | 52051282 | protein\_coding |
| ENSDARG00000008170 | dbnla | 10 | 42530046 | 42544431 | protein\_coding |
| ENSDARG00000056167 | hspe1 | 9 | 32490271 | 32494261 | protein\_coding |
| ENSDARG00000044942 | mrpl45 | 11 | 12112578 | 12121296 | protein\_coding |
| ENSDARG00000057879 | p3h3 | 16 | 12101596 | 12115926 | protein\_coding |
| ENSDARG00000029663 | rab1ab | 13 | 24271956 | 24281511 | protein\_coding |
